# In Vitro Antidiabetic, Anti-Obesity and Antioxidant Analysis of *Ocimum basilicum* Aerial Biomass and in Silico Molecular Docking Simulations with Alpha-Amylase and Lipase Enzymes

**DOI:** 10.3390/biology8040092

**Published:** 2019-12-04

**Authors:** Zoy I Noor, Dildar Ahmed, Hafiz Muzzammel Rehman, Muhammad Tariq Qamar, Matheus Froeyen, Sarfraz Ahmad, Muhammad Usman Mirza

**Affiliations:** 1 Department of Chemistry, Forman Christian College, Lahore 54600, Pakistan; 19-26018@formanite.fccollege.edu.pk (Z.I.N.); tariqqamar@fccollege.edu.pk (M.T.Q.); 2 Institute of Biochemistry and Biotechnology, University of the Punjab, Lahore 54000, Pakistan; muzzammel.phd.ibb@pu.edu.pk; 3 Department of Human Genetics and Molecular Biology, University of Health Sciences, Lahore 54000, Pakistan; 4 RAMZ Institute of Sciences, Lahore 54000, Pakistan; 5 Department of Pharmaceutical and Pharmacological Sciences, Rega Institute for Medical Research, Medicinal Chemistry, University of Leuven, B-3000 Leuven, Belgium; mathy.froeyen@kuleuven.be (M.F.); muhammadusman.mirza@kuleuven.be (M.U.M.); 6 Department of Chemistry, Faculty of Sciences, University Malaya, Kuala Lumpur 59100, Malaysia; sarfraz.ahmad@um.edu.my

**Keywords:** *Ocimum basilicum*, α-amylase inhibitory activity, diabetes, antioxidants, molecular docking

## Abstract

The present study explored phytochemicals, porcine pancreatic α-amylase (PPA) and lipase (PPL) inhibitory activities and antioxidant potential of polar and nonpolar extracts of the leaves and flowers of *Ocimum basilicum* and the in-silico mode of interaction between these enzymes and the major chemical constituents of the herb. The hexane extract (HE) and hydro-ethanolic extract (EE) obtained sequentially were used to estimate PPA and PPL inhibitory and antioxidant activities, total phenolic content (TPC) and total flavonoid content (TFC). Chemical constituents of the essential oils and HE were determined by GC-MS (Gas Chromatography-Mass Spectrometry). For PPA inhibition, IC_50_ (µg/mL) of the extracts were 0.27–0.37, which were close to 0.24 of acarbose, while for PPL inhibition, IC_50_ (µg/mL) of the extracts were 278.40–399.65, and that of Orlistat 145.72. The flowers EE was most potent antioxidant followed by leaves EE. The leaves EE had highest TPC and TFC followed of flowers EE. The essential oil of flowers had higher estragole (55%) than linalool (37%), while the essential oil of the leaves had higher linalool (42%) than estragole (38%). The HE of the flowers contained higher estragole (42%) than linalool (23%), while of the HE of the leaves too had higher estragole (65%) than linalool (18%). The in-silico molecular docking study showed linalool and estragole to have considerable PPA and PPL binding potential, which were further investigated through molecular dynamics simulations and binding free energy calculations. The PPA and PPL inhibitory activities of *O. basilicum* extracts and their notable antioxidant potential propose the herb as a multi-target complimentary medicine for diabetes, obesity and oxidative stress.

## 1. Introduction

Diabetes mellitus, obesity and oxidative stress are associated metabolic and degenerative disorders having long-term implications. Diabetes occurs either when enough insulin is not produced (type-1) or it cannot effectively be used by the body (type-2). According to the estimates of WHO (World Health Organization), diabetes prevalence is increasing worldwide. It has risen from 4.7% in 1980 to 8.5% in 2014 for adults [1]. In 2014, 8.5% of adults had this disease, while in 2015, it caused 1.6 million deaths, and, as per the WHO projections, it will be the seventh major cause of death in 2030. In addition, diabetes is a major cause of a number of complications including blindness, heart attacks, lower limb amputation, and kidney failure [1]. Type-2 diabetes is the predominant form (around 90%) of the disease. Inhibition of pancreatic α-amylase can be effective in controlling the postprandial sugar level in the patients of type-diabetes [2]. This chronic disorder has also been found to have association with obesity [3]. Obesity that is characterized by the accumulation of massive fat in the body is generally defined by a BMI of 30 or higher [4]. It is a leading risk factor for diabetes, cardiovascular disorders and cancer. Its prevalence is increasing globally and in 2016, more than 1.9 billion adults were recognized as overweight, of which over 650 million had obesity [4]. Pancreatic lipase inhibition can be used as a strategy to control absorption of diet fat into the body [2]. Oxidative stress is another metabolic disorder. It is characterized by high levels of oxygen-based reactive oxygen species (ROS) in the body and has been found to be intricately linked to obesity and type-2 diabetes [3,4]. 

Since antiquity, plants have provided human beings remedies for various types of ailments. With the view that plants have the potential to provide safe, effective and affordable remedies, the recent decades have witnessed enormous research activity in the field of phytotherapeutics [5]. Plants contain virtually an infinite reservoir of chemical substances with a great diversity of chemical functionality. They, therefore, hold great hope for discovery of new medicines. Apart from providing single molecules for allopathic medicine, herbal products can also be used as complementary and alternative medicine [6]. 

*Ocimum* is a genus of perennial and annual herbs and shrubs belonging to the family Lamiaceae comprising of almost 200 genera and 3200 species [7]. The number of species of the genus *Ocimum* is uncertain due to several taxonomical difficulties. It may therefore have 30–160 species [8]. *Ocimum basilicum* L., or sweet basil, is an important species reputed for its medicinal properties and essential oil. Its flower has bilateral symmetry with five petals and five sepals; stem is erect, branched, solid and hairy; seeds are of oval shape with black color; leaf is simple and opposite with epidermal glands having aromatic oil [9]. In recent years, *O. basilicum* has been extensively studied for its various activities [10].

Our research group studied in the past a number of properties of the plant including the α-amylase inhibitory activity of its leaves against the enzyme from *Aspergillus oryzae*. Extracts were obtained in hexane, chloroform and methanol through extraction by cold and hot extraction [11,12]. The present project was planned to compare polar and nonpolar extracts of the leaves and flowers of the herb for porcine pancreatic α-amylase and lipase inhibitory activities and antioxidant potential, and to investigate chemical constituents of their essential oils and extracts. As the nature of bioactive compounds extracted from a plant depends on the nature of solvents employed for the extraction and the extraction methods, hexane and hydro-ethanol were used as solvents for extraction, which was carried out through Soxhlet apparatus [13]. 

In-silico molecular docking is a rapidly emerging field to understand and predict possible mode of interaction between a ligand and a target biomolecule [14,15,16,17,18], while molecular dynamics simulations presented a plausible way to estimate the dynamic stability and interaction energetics of a protein–ligand complex [18,19,20,21,22,23,24]. It was, therefore, planned to conduct docking analysis of the major constituents of *O. basilicum*, linalool and estragole, with alpha-amylase and lipase together with their inhibitors.

## 2. Materials and Methods

### 2.1. Chemicals Used

The solvents used for extraction of plant material were of HPLC grade. Tris buffer were purchased from Merck (Darmstadt, Germany), porcine pancreatic lipase, ascorbic acid, starch, acarbose, DPPH (2,2-diphenyl-1-picrylhydrazyl), PNPP (*p*-nitrophenyl palmitate) and orlistat were bought from Sigma-Aldrich (USA) and porcine pancreatic α-amylase from MP Biochemicals (Illkirch, France). All the chemicals used in the experiments were of analytical grade. 

### 2.2. Collection of the Plant Material

The aerial parts of the plant *O. basilicum* L. containing leaves and flowers were collected from Sialkot (Pakistan) in April 2016. The leaves and flowers of *O. basilicum* were carefully separated from the branches. The amounts of the fresh leaves and flowers used for the study were around 450 g and 350, respectively. They were allowed to dry under shade for two weeks at room temperature to obtain 63 g leaves and 59 g flowers.

### 2.3. Preparation of Extracts

Each of the dried parts was crushed and ground to afford a fine powder, which was used to prepare, sequentially, hexanic and hydro-ethanolic extracts. Soxhlet extraction method was employed to prepare extracts. A weighed amount of each part (50 g) was packed in a filter paper thimble and extracted first with hexane (200 mL) for 5 h at 67–69 °C to obtain nonpolar chemical constituents. After this, the extraction of the defatted material was conducted with hydro-ethanol (200 mL; 80% ethanol + 20% distilled water) for 5 h at 78–80 °C to afford polar substances.

The extracts so obtained were concentrated on a rotary evaporator (R-210, Buchi, Flawil, Switzerland). They were weighed, and percent yields were calculated. These extracts were used for GC-MS analysis and bioactivities. 

### 2.4. Extraction of Essential Oil by Hydro-Distillation Method

To extract essential oil, fresh leaves and flowers of *O. basilicum* were used. Clevenger-type apparatus was used for extraction. Each of the part (50 g) was crushed and placed in the distillation flask of the apparatus. Distilled water (250 mL) was added. The apparatus was assembled, and extraction was conducted on a heating mantle for about 3 h. The essential oil obtained from each of the parts was subjected to GC-MS analysis for the identification of its constituents. 

### 2.5. Determination of α-Amylase Inhibitory Activity

Porcine pancreatic α-amylase inhibitory activities of the polar and nonpolar extracts of the leaves and flowers of *O. basilicum* were assessed according to a reported protocol [25]. Briefly, the plant solutions (0.05–0.5 µg/mL) were prepared in DMSO. α-Amylase (1 mg) was dissolved in phosphate buffer (100 mL, 20 mM, pH 6.9) to prepare the enzyme solution. The enzyme solution (0.5 mL) was mixed with an extract solution (0.5 mL). This mixture was incubated at 25 °C for 30 min. After this, 1 mL starch solution (0.5% w/v in distilled water) was added in the reaction mixture and again incubated at 25 °C for 3 min. After this, 1 mL DNS (3,5-dinitrosalicylic acid) solution was added, and mixture was heated in a water bath at 85 °C for 15 min. After this, 9 mL distilled water was added in the mixture. The absorbance was recorded at 540 nm. The percentage inhibitory activity was estimated according to the equation given below (Equation (1)):

(1) $$\%\;Inhibitory\;activity=\left[\frac{A_{c}-\;A_{s}\;}{A_{c}}\right]\times 100$$

where A_c_ and A_s_ are absorbance of negative control and the test sample, respectively. For blank, DNS solution was added before the addition of starch solution. For negative control, DMSO replaced the sample. Acarbose was used as a positive control. 

### 2.6. Determination of Lipase Inhibitory Activity

Lipase inhibitory activity of the plant extracts was estimated as per a reported protocol [26]. A stock solution of each of the plant extracts was prepared by dissolving 0.06 g of it in 100 mL DMSO. From it, further dilutions were prepared ranging from 25–600 µg/mL. *p*-Nitrophenyl palmitate (PNPP) was used as a substrate. Its solution was prepared in 2-propanol (0.1132 g per 100 mL). Orlistat was used as a positive control. Its stock solution and serial dilutions were prepared in the same manner as the plant extract solution were prepared. To prepare the lipase enzyme solution, 1 mg of the enzyme was mixed in 100 mL tris buffer (pH 8). The mixture was stirred for about 10 min, and the clear supernatant was recovered. The denatured enzyme was used as a blank, which was prepared by boiling lipase. Two test tubes were labeled as sample and negative control. In the sample test tube, lipase solution (0.5 mL) was put in a test tube to which a plant sample or standard solution (1 mL) was added. The mixture was shaken and incubated at 37 °C for 30 min in an incubator. After incubation, 1 mL of substrate solution was added to it. The reaction mixture was incubated for 2 h at 37 °C. After that, absorbance was recorded at 410 nm. In the negative control test tube, lipase solution (0.5 mL) was taken in a test tube. Buffer (1 mL) was again added (to keep the volume constant in all sets). The mixture was incubated at 37 °C for 30 min. After that, 1 mL substrate solution was added to it. The reaction mixture was incubated for 2 h at 37 °C. After that absorbance was noted at 410 nm. The percent inhibition was calculated using Equation (1).

### 2.7. Digestive System Simulation Studies for α-Amylase Inhibitory Activity

Pancreatic α-amylase activity takes place in the first part of the small intestine. Before a drug or herbal product reaches there, it must pass through the stomach. In order to have an idea of the possible effect of the gastric juice on the efficacy of the drug, we simulated the gastric system. For this purpose, a reported method was used with some modification [27]. The composition of the simulated gastric system is given in Table 1.

Each of the hydro-ethanolic extracts of leaves and flowers of *O. basilicum* (0.5 g) was separately mixed with simulated gastric juice (5 mL) and stirred for about two hours. After simulation, α-amylase inhibitory activity was evaluated. 

### 2.8. DPPH Radical Scavenging Assay

The DPPH radical scavenging potential of the polar and nonpolar extracts of leaves and flowers of *O. basilicum* were determined as per the reported assay [28]. Each of the sample solutions contained 10 mg of the respective plant extract in 50 mL methanol. Ascorbic acid was used as a positive control. The given plant sample was mixed with the DPPH working solution. After a 30-min incubation, the absorbance was noted at 517 nm. The blank had methanol in place of the plant extract. The percent free radical scavenging activity of a sample was calculated by using Equation (1). 

### 2.9. Determination of Total Phenolic Content

The total phenolic contents (TPC) of the extracts of the leaves and flowers of *O. basilicum* were determined according to a reported method using Folin–Ciocalteu reagent [29]. The commercially available Folin–Ciocalteu reagent was used. Gallic acid was used as a positive control, whose serial dilutions were made with concentrations 50–500 mg/L in distilled water.

One milliliter (1 mL) plant sample (1 mg per mL of methanol) was mixed with distilled water (4.6 mL). Then, 1 mL Folin–Ciocalteu reagent was mixed. After 30 min, 3 mL Na_2_CO_3_ solution (2.5 M) was added. After an incubation of 30 min, absorbance was measured at 765 nm. The TPC was expressed as μg/mL of GAE, which was converted to μg GAE per g of sample. The blank had methanol in place of sample.

### 2.10. Determination of Total Flavonoid Content 

The total flavonoid contents (TFC) of leaves and flowers of *O. basilicum* were determined as per a reported colorimetric method [29]. In the method, aluminum chloride form coordination complex with flavonoids. The flavonoid rutin was used as a standard and its serial dilutions were made in methanol with concentrations 250–750 mg/L to construct a calibration curve.

A sample (300 μL; 1 mg/mL) was dissolved in methanol (3.4 mL). In the solution so obtained 0.5 M NaNO_2_ (150 µL) and 0.3 M AlCl_3_ solution (150 µL) were added. After 5 min, 1 mL NaOH (1 M) was mixed. Its absorbance was recorded at 506 nm. The TFC was expressed as μg/mL of RE which was converted to μg RE per g of sample. The blank contained equal volume of methanol in place of sample.

### 2.11. Identification of Chemical Compounds by GC-MS 

The GC-MS analysis of the essential oils and hexane extracts of the leaves and flowers of *O. basilicum* was carried out to identify their chemical constituents. The GC-MS equipment was Agilent 7890 A/5975C (Agilent, Santa Clara, CA, US). The GC had an HP-5 column (length 30 m with internal diameter 250 µm); 1.0 µl sample was injected in a split mode (1:100); oven temperature was 35–320 °C; injector temperature 180 °C. MS worked at 70 eV; scan time 1.5 second and mass range 50–600 amu. NIST 05 spectral library was used for identification of chemical compounds. 

### 2.12. Molecular Docking Analysis and Molecular Dynamics Simulations

The identified potential compounds after GC-MS analysis were used for molecular docking studies to analyze the most favorable conformation and binding affinity with respective targets. X-ray resolved crystal structures of porcine pancreatic α-amylase (PPA) complexed with a carbohydrate inhibitor, acarbose (PDB ID: 1OSE) and lipase (PPL) complexed with methoxy-undecylphosphinic acid (MUP) (PDB ID: 1LPB) were obtained from Protein Data Bank [30]. Before docking, protein structures were minimized and optimized using the same protocol as described elsewhere [14,23,24,31]. For Molecular docking, AutoDock Vina was employed, which follows a gradient optimization method in its local optimization procedure to rank the ligands based on empirical binding free energy (ΔG in kcal/mol) function [32,33]. The overall docking procedure was first evaluated by re-docking of the co-crystallized inhibitor into the respective binding site of PPA and PPL, and root-mean-square-deviations were estimated. The best protein/ligand complexes were analyzed through UCSF Chimera 13.1 [34]. To better understand the dynamic stability of protein/ligand complex, a 20ns MD simulations was performed and backbone Cα stability was examined. The AMBER16 simulation package was employed for molecular dynamic simulations which uses the AMBER ff99SB force field [35,36]. The same stepwise energy minimization and equilibration protocol was utilized as described in previous studies [18,21,23,24]. The solvated system with explicit water molecular (TIP3 system) was submitted to a production run of 20ns at constant 300 K temperature and 1 bar pressure. The RMSD between the Cα atoms of the protein and all the atoms of the ligand was calculated to evaluate the protein/ligand complex stability. The MD simulation trajectory was analyzed using the CPPTRAJ module [37]. The most representative conformation of the complex was obtained from MD clustering and was analyzed for molecular interactions. The binding free energy calculations were computed through molecular mechanics/generalized born solvent area (MM/GBSA) method implemented in AMBER 16 [38] to gain rational insights into the electrostatic and van-der-Waals (vdw) contributions.

### 2.13. Statistical Analysis

The inhibitory concentration (IC_50_) and effective concentration (EC_50_) values were calculated from linear regression analysis of the percent activity as a function of concentration by using The Microsoft Excel 2010, and the analysis of variance (ANOVA) was applied on the data of response variables to determine the significant difference (P < 0.05) using Minitab 17 statistical software (Minitab Ltd., Coventry, UK).

## 3. Results and Discussion 

### 3.1. Extraction Yield 

In the present research, fresh leaves and flowers of *O. basilicum* were used, which were crushed, ground, and extracted first with hexane to remove nonpolar and fatty material and then with hydro-ethanol to capture polar substances. Hot extraction method was used employing Soxhlet apparatus. Percent yields of extracts were calculated based on the dried ground material. The hexanic and hydro-ethanolic extracts of leaves had 1.84% and 5.28% yield, respectively; while, hexanic and hydro-ethanolic extracts of flowers showed 2.02% and 2.4% yield, respectively. Thus, leaves had much higher content of polar substances than flowers.

### 3.2. Alpha-Amylase Inhibitory Activity

**Figure 1:**
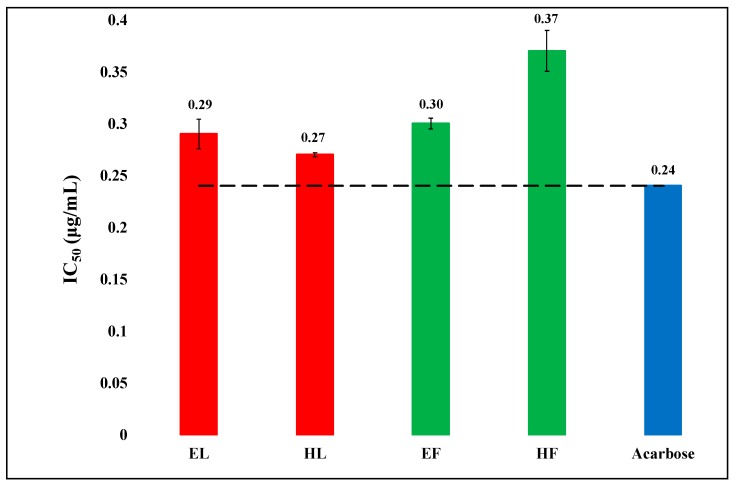
The comparison of α-amylase inhibitory activity (IC_50_) in hexanic (H) and hydro-ethanolic (E) extracts of flowers (F) and leaves (L) of *Ocimum basilicum* with the standard acarbose. The reference (dash) line shows the difference in activities from standard acarbose and the results are significantly different with P < 0.05. Vertical segment at each bar peak is the Sdev.

The α-amylase inhibitory activities of hexanic and hydro-ethanolic extracts of leaves and flowers of *O. basilicum* were evaluated against porcine pancreatic α-amylase. The results are exhibited in Figure 1. The extracts exhibited excellent α-amylase inhibitory activities. Their IC_50_ (µg/mL) values were in the range of 0.27–0.37 while that of acarbose was 0.24.

### 3.3. Lipase Inhibitory Activity

**Figure 2:**
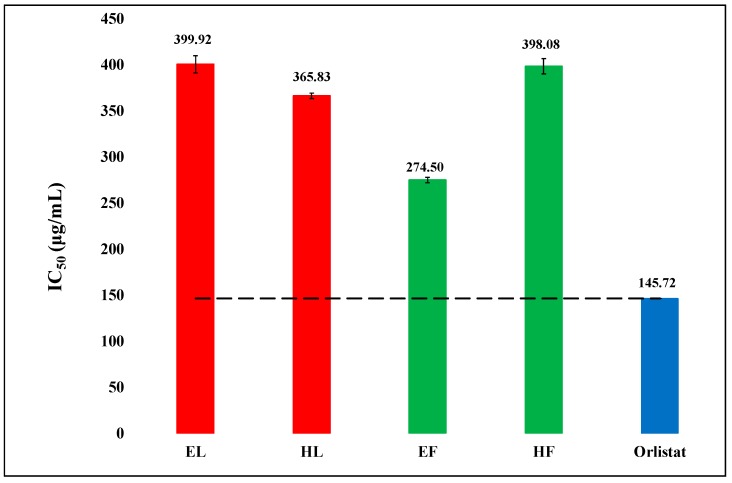
The comparison of lipase inhibitory activity (IC_50_) of hexanic (H) and hydro-ethanolic (E) extracts of flowers (F) and leaves (L) of *Ocimum basilicum* with the standard orlistat. The reference (dash) line represents the difference in activities of extracts from standard orlistat and the outcomes were observed highly significant (P < 0.05). Vertical segment at each bar peak is the Sdev.

Lipase inhibitory activities of hexanic and hydro-ethanolic extracts of leaves and flowers of *O. basilicum* were assessed using porcine pancreatic lipase, and the results are exhibited in Figure 2. The activities were notable though the extracts were less potent than the standard drug orlistat used in the study. The IC_50_ (µg/mL) values of the extracts were in the range of 274.50–399.92, while that of orlistat was 145.72. The flowers hydro-ethanolic extract (EF) was drastically more active than the other extracts. It was followed by hexanic leaves extract (HL). 

### 3.4. Effect of Gastric Juice on α-Amylase Inhibitory Activity

**Figure 3:**
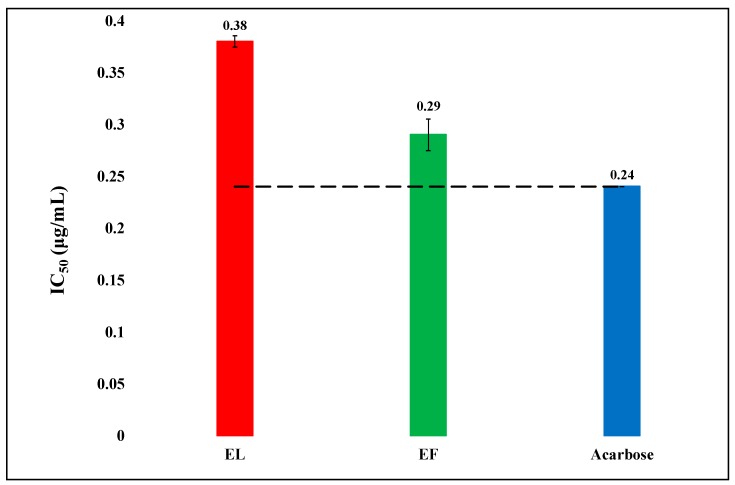
The comparison of α-amylase inhibitory activity (IC_50_) of hydro-ethanolic (E) extracts of flowers (F) and leaves (L) of *Ocimum basilicum* with the standard acarbose after passing through a simulated digestive system. The refence (dash) line shows a significant difference (P < 0.05) among the activities. Vertical segment at each bar peak is the Sdev.

Effect of the gastric juice on the α-amylase inhibitory activity of the herbal samples were studied using simulated gastric juice, and the results are displayed in Figure 3. The flowers extract remained unaffected and was more active than that of the leaves. The simulated gastric juice affected the α-amylase inhibitory activity of the hydro-ethanolic extract of leaves considerably. 

### 3.5. DPPH Free Radical Scavenging Activity

**Figure 4:**
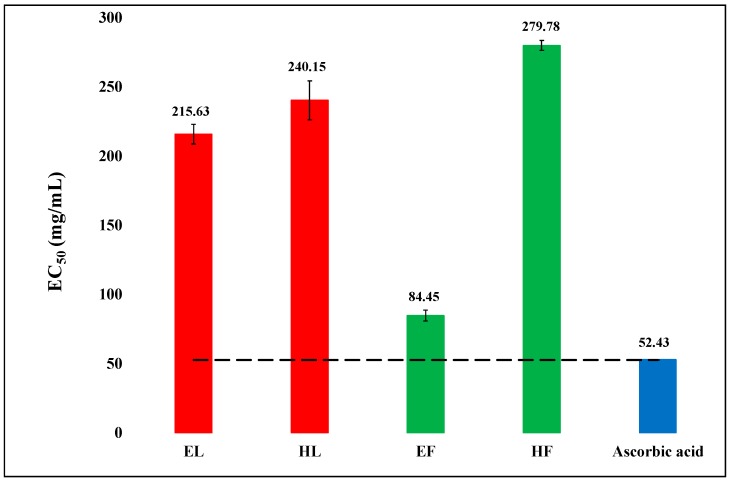
The comparison of DPPH free radical scavenging activity (EC_50_) of hexanic (H) and hydro-ethanolic (E) extracts of flowers (F) and leaves (L) of *Ocimum basilicum* with the standard ascorbic acid. The reference (dash) line indicates the significance difference (P value < 0.05) in the scavenging efficiency of the extract in comparison to ascorbic acid. Vertical segment at each bar peak is the Sdev.

The DPPH free radical scavenging potential of hexanic and hydro-ethanolic extracts of leaves and flowers of *O. basilicum* were assessed using the well-known DPPH assay. The results are exhibited in Figure 4.

The activity was dose-dependent. The hydro-ethanolic extract of flowers was most potent followed by hydro-ethanolic extract of leaves. The hexanic extract of flowers was least active.

### 3.6. Total Phenolic and Total Flavonoid Contents 

**Figure 5:**
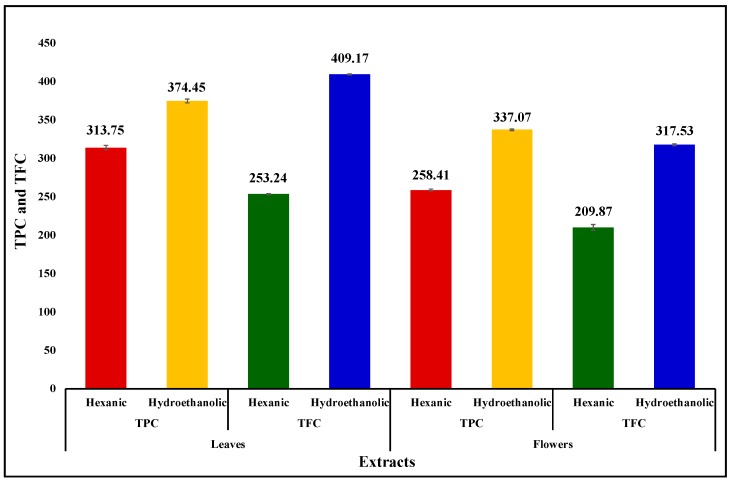
The comparison in total phenolic content (TPC) and total flavonoid content (TFC) present in the Hexanic (H) and Hydro-ethanolic (E) extracts of leaves (L) and flowers (F) of *Ocimum basilicum* in terms of gallic acid and rutin equivalents, respectively. Vertical segment at each bar peak is the Sdev.

The TPC and TFC of the extracts of leaves and flowers of *O. basilicum* were evaluated and the results are compared and presented in the form of a bar graph in the Figure 5. 

The following trends in the respective contents were observed: TPC: E leaves > E flowers > H leaves > H flowers; TFC: E leaves > E flowers > H leaves > H flowers.

Thus, both the contents exhibited similar trends. Polar extracts had higher phenolics and flavonoids whereas leaves had higher amounts than flowers. 

### 3.7. Identification of Chemical Compounds 

GC-MS screening of the essential oils and extracts of flowers and leaves of *O. basilicum* was resulted in the identification of several compounds in them. The results are displayed in Table 2, Table 3, Table 4 and Table 5. The major components of the essential oil extracted from the flowers included estragole (p-allylanisole or methyl chavicol; 54.99%) 3,7-dimethyl-1,6-octadien-3-ol (linalool; 36.88%), gamma-cadinene (2.41%), and camphor (1.394%). The chemical constituents of the essential oil obtained from the leaves included linalool (42.229%), estragole (38.022%), gamma-cadinene (4.089%), Z-ocimene (3.879%), eucalyptol (2.980%), camphor (2.093%), and α-gergamotene (1.348). Chemical constituents of hexanic extract of the flowers included estragole (41.618%), linalool (23.244%), beta-elemene (5.585%), delta-cadinol (6.776%), D-germacrene (4.069%), gamma-cadinene (2.301%), and caryophyllene (2.263%). Chemical constituents of the hexanic extract of the leaves included estragole (64.623%), linalool (17.623%), cadinol (3.474%), α-bergamotene (2.848%), and phytol (2.652%).

### 3.8. Molecular Modeling Interpretations 

**Figure 6:**
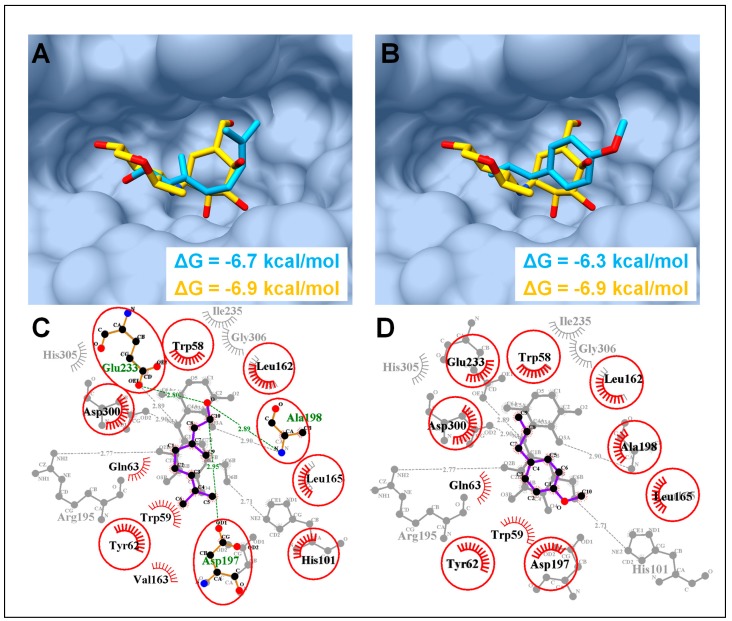
Molecular docking analysis of porcine pancreatic α-amylase (PPA). (**A**) Molecular surface representation of PPA complexed with co-crystalized acarbose (yellow sticks) and linalool (blue sticks). (**B**) PPA complexed with estragole with same color code as in (A). (**C**) 2D interaction analysis of linalool superimposed on co-crystalized acarbose (PDB ID: 1OSE). Hydrogen-bonded residues are in sticks, hydrophobic interacted residues are with spoked arcs while common residues in both complexes are highlighted with red circles. (**D**) 2D interaction analysis of estragole superimposed on co-crystalized acarbose. Same color code as in (C).

**Figure 7:**
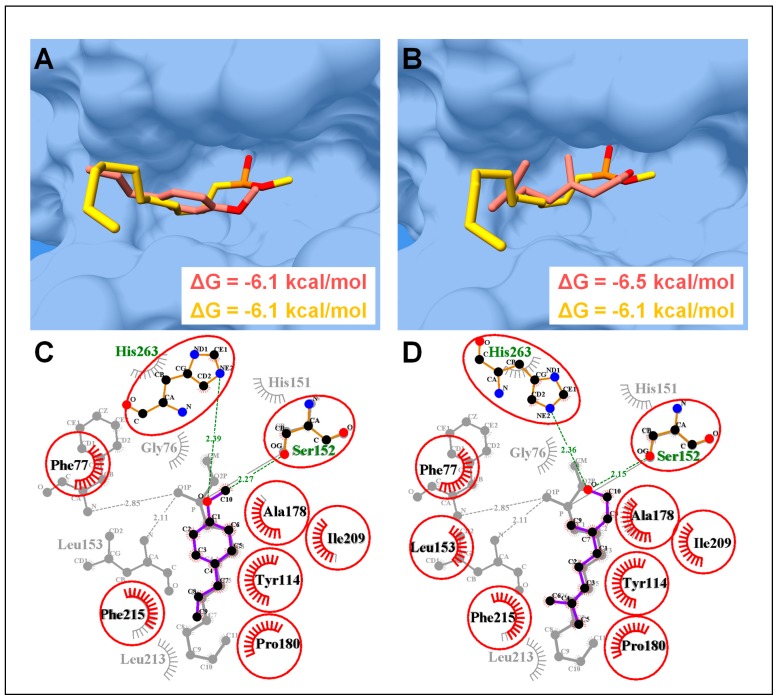
Molecular docking analysis of porcine pancreatic lipase (PPL). (**A**) Molecular surface representation of PPL complexed with co-crystalized MUP (yellow sticks) (PDB ID: 1PBL) and estragole (blue sticks). (**B**) PPL complexed with linalool. Same color code as in (**A**). (**C**) 2D interaction analysis of estragole superimposed on co-crystalized MUP. Hydrogen-bonded residues are in sticks, and hydrophobic interacted residues are with spoked arcs while common residues in both complexes are highlighted with red circles. (**D**) 2D interaction analysis of linalool superimposed on co-crystalized MUP. Same color code as in (**C**).

Based on the GC-MS analysis, linalool and estragole were found as major chemical constituents which presumably contribute significantly in PPA and PPL inhibitory activities. Molecular docking revealed the conformation of estragole and linalool in agreement with the co-crystallized inhibitors of PPA and PPL. Hence, linalool and estragole were found to have consensus binding mode with co-crystalized acarbose with PPA and MUP (methoxyundecyl phosphinic acid) with PPL [39]. Furthermore, linalool showed more favorable binding energy (ΔG = −6.7 and −6.5 kcal/mol) as compared to estragole (ΔG = −6.3 and −6.1 kcal/mol) with PPA and PPL, respectively (Figure 6 and Figure 7). 

In PPA complexes, both compounds were able to form the favorable conformation inside the α-amylase active site (Figure 6A,B), where Asp197, Glu233, and Asp300 form the catalytic triad [40]. Among both compounds, linalool was found to interact with catalytic triad through H-bonds with the side chain oxygen atom of Asp197 (2.95Å), and Glu233 (2.80Å), alongside an additional H-bond with neighboring residue Ala198 (2.89Å) (Figure 6C). Meanwhile, no H-bond in an atomic radius of 3Å was found in estragole-PPA complex, although a number of consensus hydrophobic interactions were found in both complexes with residues, Trp58, Tyr62, Leu165, and Leu162 (Figure 6C,D). 

In case of PPL complexes, both compounds were found to interact deep inside the enzyme active site (Figure 7A,B), where His263, Asp176, and Ser152 make a catalytic triad, which actively participates in lipolytic activity [41]. In-depth molecular interaction elucidated the role of terminal oxygen atom of both the compounds in forming H-bonds (atomic distance <3Å) with the side chain oxygen and the nitrogen atom of Ser152 (OG) and His263 (NE2), respectively. Together with H-bonds, both compounds were able to interact through a number of hydrophobic interactions, which included, Phe77, Tyr114, Ala178, Pro180, Ile209, and Phe215 (Figure 7C,D). 

**Figure 8:**
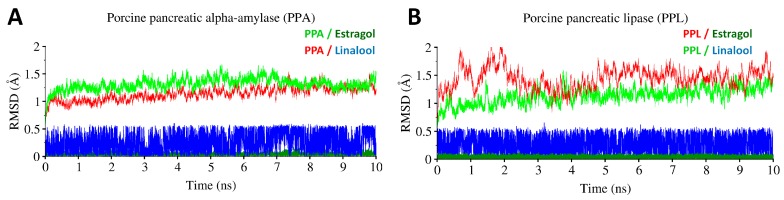
Molecular dynamics simulations of estragol and linalool in complex with PPA and PPL. (**A**) Root mean square deviation (RMSD) trajectories in time-dependent manner (ns) of PPA in complex with estragole and linalool. (**B**) Root mean square deviation (RMSD) trajectories in time-dependent manner (ns) of PPL in complex with estragole and linalool.

The complexes were further investigated through 10ns MD simulation to assess the protein dynamic stability and energy contributions of ligands in complex with PPA and PPL. The RMSD trajectory of all simulated complexes are displayed in Figure 8. Both compounds in complex with PPA showed stability conformation throughout MD simulation. The Cα-backbone stability of PPA with bound ligands remained the same and did not show extreme fluctuations, whereas the Cα-backbone stability of PPL fluctuated in the start and remained converge in case of linalool, but bound estragol induced slight fluctuations in the protein’s backbone Cα atoms. 

To gain further insight into the binding affinities, MM-GBSA analysis was performed to calculate the contributions of electrostatic (ΔE_ele_), van der Waals (ΔE_vdw_), and solute-solvent energies (ΔG_np_ and ΔG_p_) towards total binding free energy (ΔG_tol_) as tabulated in Table 6. In PPA, the overall binding free energies (ΔG_tol_) of estragole and linalool were −20.34 and −16.75 kcal/mol, which showed fairly good binding affinities as compared to PPL (ΔG_tol_ = −15.89 kcal/mol for estragole and −13.74 kcal/mol for linalool). For both proteins, major total free energy contributions to the ligand binding were due to the favorable van der Waals interactions (ΔE_vdw_) as compared to electrostatic interactions (ΔE_ele_) as shown in Table 6. Nonpolar solvation energies (ΔG_np_) also contributed towards ligand binding, while polar solvation energy (ΔGp) revealed unfavorable contributions.

As is evident from the results of the in-vitro study, *O. basilicum* extracts showed remarkable α-amylase inhibitory activity in a dose dependent manner, which was almost equivalent to the positive control acarbose. The results revealed that leaves extracts were more active than those of the flowers. 

After digestive system simulation, IC_50_ of EF and EL were 0.29 and 0.38, respectively, while their IC_50_ without simulation were 0.3 and 0.29, respectively. Thus, simulation had almost no effect on the efficacy of EF, but it drastically affected that activity of EL. Many studies have shown the herb to have α-amylase inhibitory activity [42]. Recently, Malapermal et al. evaluated the α-amylase inhibitory activity of aqueous, 60% ethanol and 70% ethanolic extracts of the leaves of *O. basilicum*, and the extracts showed activity almost comparable to acarbose [43]. The present study showed the similar results as presented by Malapermal et al.

The results, therefore, suggest that *O. basilicum* should inhibit human pancreatic α-amylase in the small intestine suppressing the digestion of carbohydrates and thereby controlling the entry of glucose into human body. This would keep the postprandial glucose level in a limit. 

As is known, the pancreatic lipase hydrolyses lipids into glycerol and fatty acids. The inhibition of this enzyme will, therefore, suppress the conversion of lipids into the products that can be absorbed into the body. Hence, the drugs or herbal products having lipase inhibitory activity provide a strategy to control obesity. The common drug orlistat follows the same mechanism but it has a number of side effects such as steatorrhoea, oily spotting, and fecal issues [26]. Plant based inhibitors of the enzymes would have many advantages over the synthetic drugs, such as safety, efficacy and affordability and it would be safer for a patient to use plant-based drug than a synthetic drug. The extracts of the leaves and flowers of *O. basilicum* exhibited considerable lipase inhibitory activity. The hydro-ethanolic extract of the flowers was noticeably more active. Our results corroborate the findings of previous studies. Recently, Irondi et al. (2016) studied ethyl acetate extract of the leaves of *O. basilicum* from Nigeria [13]. The extract showed good lipase inhibitory activity, which was, however, less than orlistat. These studies establish the efficacy of this culinary herb to control obesity through controlling the lipid digestion in the alimentary canal.

A number of authors studied antioxidant properties of the essential oils and extracts of this plant [26]. Most studies explored either its leaves or areal parts as a whole. No comparison of leaves and flowers have been reported so far. The chemical compound DPPH is a stable free radical that is commonly used to estimate radical scavenging ability of a drug or a herbal product [44]. It is considered to follow both the single electron transfer (SET) as well as hydrogen atom transfer (HAT) mechanism, thus, giving an estimation of antioxidants having potential to follow both or either of the mechanisms. The extracts of *O. basilicum*, in the present work, showed considerable potency to scavenge free radicals alluding to the possible application of the plant to combat degenerative diseases. The EC_50_ value shown by hydro-ethanolic extract of flowers and leaves of the plant was much lower indicating a higher antioxidant potential. The order of radical scavenging activity was ascorbic acid > EF > EL > HL > HF. The EF was noticeably more active than the other extracts.

The studies showed hydro-ethanol as a better solvent to extract chemical substances with antioxidant and radical scavenging potential. There was a correspondence between the lipase inhibitory activity and the antioxidant activity, referring to a possibility that the compounds having high antioxidant activity may also have high lipase inhibitory activity. The hydro-ethanolic extract of flowers was the most active antioxidant as well as lipase inhibitor. 

The bioactivities of the plant extracts can at least partly be explained on the basis of their phenolic and flavonoid contents. As Figure 1 and Figure 5 show, there is some correspondence in the TPC and TFC and α-amylase inhibitory activity. The hexanic leaves are an exception, which is the most potent inhibitor of the enzyme, but very low in both TPC and TFC. As Figure 2 displayed, the order of the lipase inhibitory activity was EF > HL > HF > EL; only partial correspondence can be postulated between TPC/TFC and lipase inhibitory activity. As can be seen in Figure 4, the order of radical scavenging activity of the extracts was EF > EL > HL > HF; there is an excellent correspondence between TPC/TFC and radical scavenging activity. The polar extracts with higher TPC and TFC were more potent antioxidants than the non-polar extracts.

Estragole and linalool were two major constituents of the essential oils and hexanic extracts of flowers and leaves of *O. basilicum*. Composition of the essential oil from the herb has been studied by many research groups around the world and produced diverse results. Zheljazkov et al. (2008) evaluated the oil composition of 38 accessions of *O. basilicum* grown in Mississippi and divided, based on the oil composition, the accessions into seven groups: (1) high-linalool chemotype, (2) linalool-eugenol chemotype, (3) methyl chavicol chemotype, (4) methyl chavicol-linalool chemotype, (5) methyl eugenol-linalool chemotype, (6) methyl cinnamate-linalool chemotype, and (7) bergamotene chemotype [45]. According to this classification, the plant used in the present study belonged to the methyl chavicol-linalool chemotype. Similar diverse findings have been reported by another researcher as well [46]. Fatope et al. (2008) compared the essential oil composition of the leaves and floral parts of the plant belonging to linalool chemotype [46]. The flowers had higher content of linalool than the leaves. Recently, Tshilanda et al. (2016) investigated the essential oil composition of the leaves of *O. basilicum* from Congo, which came out to be the methyl chavicol–linalool chemotype [47]. Methyl chavicol was the most abundant component (35.72%) followed by linalool (21.25%) and then epi-α-cadinol (8.02%), α-bergamotene (6.56%), eugenol (4.60%), 1,8-cineole (4.04%), germacrene-D (2.06%), thymol (1.64%), and (E)-citral (1.55%). The results of our study were in consonance with them. In the current work, we have not only compared essential oil from leaves and flowers, but hexanic extracts of the both parts as well. 

Molecular docking coupled with MD simulations is an important method to elucidate the mode of interaction of ligands with specific targets, such as enzymes, and to comprehend the enzyme inhibition mechanisms [18,21,23,24,48]. Our in-silico molecular modeling study revealed a favorable binding affinity of linalool and estragole, the two major constituents of this plant, with PPA and PPL (Figure 6 and Figure 7) and both compounds showed a similar conformation and interactions pattern as reported with their co-crystalized inhibitors. For example, hydroxyethyloxy)tri(ethyloxy)octane and MUP complexed with PPL were reported to interact with the catalytic triad (His263, Asp176, and Ser152) through number of H-bonds and MUP was covalently bound to Ser152 [39,49]. However, acarbose was found to interact with three catalytically important residues Asp197, Glu233, and Asp300, which are reported as conserved catalytic fold for several families of glycosyl hydrolases [41]. Both compounds were found to interact with these catalytically important residues and exhibited favorable binding affinities as calculated through MMGBSA module, this speculated that these compound contributed significantly in enzyme inhibition activities. Since it is difficult to categorize a single compound responsible for the whole inhibitory activities, we can speculate, based on the experimental and in-silico results, that PPA and PPL inhibitory activities of *O. basilicum* hexane and hydro-ethanolic extract probably has a good share of the synergistic effect of these active constituents

## 4. Conclusions

The hexanic and hydro-ethanolic extracts of leaves and flowers of *Ocimum basilicum* were evaluated for antioxidant, antidiabetic and anti-obesity potential using a number of in-vitro assays. Both the parts exhibited α-amylase inhibitory activity almost equivalent to the drug acarbose. The anti-lipase activity was also notable though it was somewhat less than the standard drug orlistat. The major chemical compounds identified by GC-MS included estragole, linalool, γ-cadinene, D-germacrene, and β-elemene. The in-silico docking studies showed linalool and estragole to have exploitable inhibitory activities against both the enzymes.

## Figures and Tables

**Table 1:** Composition of simulated gastric juice for pancreatic alpha-amylase inhibitory study.

Solutions for Gastric Juice	Amount
Distilled water	250 mL
NaCl	1.375 g
NaHCO_3_	0.53 g
NH_4_Cl	0.153 g
Urea	0.045 g
Concentrated HCl	3.26 mL
CaCl_2_·H_2_O	0.20 g
KCl	0.41 g
NaH_2_PO_4_	0.133 g

**Table 2:** Chemical constituents of the essential oil obtained from the flowers of *O. basilicum* as identified by GC-MS analysis.

Peak	RT (min)	Compound Name	Relative Percentage (%)
1	8.688	Eucalyptol	0.367
2	9.095	3,7-Dimethyl-1,3,6-octatriene,	0.989
3	10.506	3,7-Dimethyl-1,6-octadien-3-ol (linalool)	36.880
4	11.126	Camphor	1.394
5	12.264	Estragole (methyl chavicol)	54.989
6	14.915	Cyclohexane, 1-ethenyl-1-methyl-2,4-bis(1-methylethenyl)-, [1S-(1α,2β,4β)]- (β-Elemene)	0.301
7	15.339	Caryophyllene	0.395
8	15.535	Azulene,1,2,3,4,5,6,7,8-octahydro-1,4-dimethyl-7-(1-methylethenyl)-,[1S-(1α,4α,7 α)]- (α-Guajene)	0.210
9	16.155	1,6-Cyclodecadiene, 1-methyl-5-methylene-8-(1-methylethyl)-,[s-(E,E)]- (D-Germacrene)	0.850
10	16.342	γ-Elemene	0.178
11	16.444	Azulene, 1,2,3,5,6,7,8,8a-octahydro-1,4-dimethyl-7-(1-methylethyenyl)-,[1S-(1α,7 α,8aβ)]- (α-Bulnesene)	0.204
12	17.463	Caryophyllene Oxide	0.110
13	17.820	Carotol	0.316
14	18.160	Napthalene, 1,2,3,4, 4a,5,6,8a-octahydro-7-methyl-4-methyelene-1-(1-methylethyl)-, (1α,4aα,8aα)- (γ-cadinene)	2.409

**Table 3:** Chemical constituents of the essential oil obtained from the leaves of *O. basilicum* as identified by GC-MS analysis.

Peak	RT (min)	Compound Name	Relative Percentage (%)
1	7.394	Bicyclo[3.1.1]heptane,6,6-dimethyl-2-methylene-, (1S)-	0.221
2	7.768	β-Myrcene	0.413
3	8.736	Eucalyptol	2.980
4	9.153	1,3,6-Octatriene, 3,7-dimethyl-, (Z)- (Z-Ocimene)	3.879
5	10.597	3,7-dimethyl-1,6-octadien-3-ol (Linalool)	42.229
6	11.226	Camphor	2.093
7	12.279	Estragole	38.022
8	13.468	Acetic acid, 1,7,7-trimethyl-bicyclo[2.2.1]hept-2-yl ester	0.266
9	14.938	Cyclohexane, 1-ethenyl-1-methyl-2,4-bis(1-methylethenyl)-,[1S-(1α,2β,4β)]-	0.241
10	15.133	Benzene, 1,2-dimethyoxy-4-(2-propenyl)-	0.380
11	15.380	Caryophyllene	1.238
12	15.507	Bicyclo[3.1.1]hept-2-ene,2,6-dimethyl-6-(4-methyl-3-pentenyl)- (α-Bergamotene)	1.348
13	16.170	1,6-Cyclodecadiene, 1-methyl-5-methylene-8-(1-methylethyl)-,[s-(E,E)]-	0.960
14	16.357	γ-Elemene	0.273
15	16.578	Napthalene, 1,2,3,4, 4a,5,6,8a-octahydro-7-methyl-4-methyelene-1-(1-methylethyl)-, (1α,4aβ,8aα)-	0.870
16	17.818	Cubenol	0.494
17	18.183	Napthalene, 1,2,3,4, 4a,5,6,8a-octahydro-7-methyl-4-methyelene-1-(1-methylethyl)-, (1α,4aα,8aα)- (γ-Cadinene)	4.089

**Table 4:** Chemical constituents of hexane extract of the flowers of *O. basilicum* as identified by GC-MS analysis.

Peak	RT (min)	Compound Name	Relative Percentage (%)
1	10.560	3,7-dimethyl-1,6-Octadien-3-ol (Linalool)	23.244
2	12.242	Estragole	41.618
3	14.833	Cyclohexane, 1-ethenyl-1-methyl-2,4-bis(1-methylethenyl)-	0.325
4	15.003	Cyclohexane, 1-ethenyl-1-methyl-2,4-bis(1-methylethenyl)-,[1S-(1α,2β,4β)]- (β-Elemene)	5.585
5	15.394	Caryophyllene	2.263
6	15.521	Bicyclo[3.1.1]hept-2-ene,2,6-dimethyl-6-(4-methyl-3-pentenyl)- (α-Bergamotene)	1.191
7	15.581	Azulene,1,2,3,4,5,6,7,8-octahydro-1,4-dimethyl-7-(1-methylethenyl)-,[1S- (1α,4α,7α)]-	0.691
8	15.827	Caryophyllene	0.527
9	15.938	1H-Cyclopenta[1,3]cyclopropa[1,2]benzene,octahydro-7-methyl-3-methylene-4-(1-methylethyl)-,[3aS-(3a α,3b.β,4β,7 α,7aS)]-	0.345
10	16.218	1,6-Cyclodecadiene, 1-methyl-5-methylene-8-(1-methylethyl)-,[S-(E,E)]- (D-Germacrene)	4.069
11	16.371	Azulene, 1,2,3,3a,4,5,6,7-octahydro-1,4-dimethyl-7-(1-methylethyenyl)-,[1R-(1α,3aβ,4α,7β)]-	0.460
12	16.473	Azulene,1,2,3,5,6,7,8,8a-octahydro-1,4-dimethyl-7-(1-methylethenyl)-,[1S-(1α,7α,8aβ)]-	1.039
13	16.609	Naphthalene, 1,2,3,4, 4a,5,6,8a-octahydro-7-methyl-4-methyelene-1-(1-methylethyl)-, (1α, 4aβ,8aα )- (γ-Cadinene)	2.301
14	17.475	Caryophyllene Oxide	0.511
15	17.849	Cubenol	0.789
16	18.231	1-Napthalenol, 1,2,3,4, 4a,7,8,8a-octahydro-1,6-dimethyl-4-(1-methylethenyl)-,[1S-(1α,4α,4aβ,8aβ)]- (delta-Cadinol)	6.776
17	18.342	α-Cadinol	0.434
18	22.827	Phytol	0.753
19	23.278	8,11,14-Eicosatrienoic acid,(Z,Z,Z)-	0.579

**Table 5:** Chemical constituents of the hexane extract of the leaves of *O. basilicum* as identified by GC-MS analysis.

Peak	RT (min)	Compound Name	Relative Percentage (%)
1	10.322	3,7-Dimethyl-1,6-octadien-3-ol (Linalool)	17.623
2	12.217	Estragole	64.623
3	14.910	Cyclohexane, 1-ethenyl-1-methyl-2,4-bis(1-methylethenyl)-,[1S-(1α,2β,4β)]- (β-Elemene)	0.996
4	15.335	Caryophyllene	1.646
5	15.479	Bicyclo[3.1.1]hept-2-ene,2,6-dimethyl-6-(4-methyl-3-pentenyl)- (α-Bergamotene)	2.848
6	16.142	1,6-Cyclodecadiene, 1-methyl-5-methylene-8-(1-methylethyl)-,[S-(E,E)]-	1.102
7	16.549	Naphthalene, 1,2,3,4, 4a,5,6,8a-octahydro-7-methyl-4-methylene-1-(1-methylethyl)-, (1α, 4aβ,8aα)- (γ-Cadinene)	1.508
8	18.138	Cadinol	3.474
9	22.785	Phytol	2.652
10	28.409	Squalene	2.472
11	30.133	Heptacosane	0.780
12	31.578	Hexatriacontane	0.732

**Table 6:** Molecular mechanics generalized born surface area (MM-GBSA) binding free energy calculation of estragole and linalool in complex with PPA and PPL.

Contributions	PPA (kcal/mol)		PPL (kcal/mol)	
	estragole	linalool	estragole	linalool
**ΔE**_**ele**_	–6.53	–4.66	–5.04	–4.12
**ΔE**_**vdw**_	–19.41	–18.58	–15.41	–14.54
**ΔE**_**MM**_	–25.94	–23.24	–20.45	–18.66
**ΔG**_**p**_	8.86	10.5	8.48	6.54
**ΔG**_**np**_	–3.26	–4.01	–3.92	–1.62
**ΔG**_**sol**_	5.6	6.49	4.56	4.92
**ΔG**_**tol**_	–20.34	–16.75	–15.89	–13.74

Note: ΔG_tol_ is the sum of molecular mechanics energy (ΔE_MM_) and solvation free energy (ΔG_sol_). Both ΔE_MM_ and ΔG_sol_ are further divided into internal energy (ΔE_int_), electrostatic energy (ΔE_ele_), and van der Waals (ΔE_vdw_) energy in the gas phase, and polar (ΔG_p_) and non-polar (ΔG_np_) contributions to the solvation free energy.
